# History of a Case in Which a Large Stone Was Extracted from the Urinary Bladder by the Rectum

**Published:** 1820-05

**Authors:** N. Barbantini

**Affiliations:** Surgeon to the Royal Hospital at Lucca.


FOR THE LONDON MEDICAL AND PHYSICAL JOURNAL.

### *History of a Case in which a large Stone was extracted from the Urinary Bladder by the Rectum.* By N. Barbantini, M.D. Surgeon to the Royal Hospital at Lucca.

A MAN, fifty years of age, whose constitution was much altered by the long sufferings he had experienced, requested that I would examine the state of his bladder, to disease of which he attributed all his pains. On a catheter being introduced, the presence of a calculus was readily discovered; and, by the resistance it made to the instrument, it appeared to be of considerable size. In order that its dimensions might be better ascertained, I passed a finger into the rectum, and then found, not without surprise, that the lower part of this intestine was almost entirely filled by a solid movable tumor of considerable bulk, covered by the paries of the two organs, that is to say, the bladder and rectum. This tumor rested on the sphincter, the weight of the stone having pressed downwards considerably the posterior part of the bladder. On running my finger over its surface, I found that it extended from one tuberosity of the ischium to the other, and the absolute impossibility of extracting it by the lateral operation became evident. The breaking such a stone in the bladder was, perhaps, neither easy nor prudent: we know that fatal accidents are the common results of such attempts, and these are often unsuccessful from the hardness of the calculus. Now I was here ignorant of the quality of the substance composing the body I had to encounter. The high operation might, it is true, be resorted to; but this method has always seemed to me to be difficult in the execution, and more uncertain than the other in its results: I determined, therefore, to practise the operation through the rectum. By this method I should divide but few parts; I should obtain a large opening with membranous, and but little irritable, parietes; I should avoid important nerves and blood-vessels: in a word, it appeared that this proceeding would be most satisfactory to my judgment, and promised the most advantage to the patient in the critical situation in which he was placed.

An unforeseen accident retarded for some days the execution of the operation. The bladder, as already stated, was forcibly pressed towards the rectum, and the stone prevented the catheter passing much beyond the prostate. I found, on passing a finger into the rectum, that the point of the instrument struck against the foreign body, and it was not possible to raise the latter or depress the former sufficiently to permit the catheter to pass along the lower and posterior part of the bladder. How, then, could membranous parts, presenting so little resistance, be cut without the guide of the groove of the sound. It was not possible to make the bistoury act on the stone itself, because of its mobility and the asperities of its surface. I had made, then, a very long canulated sound, which I proposed to pass into the bladder through the first incision, and which I hoped to be able to direct with more facility than a catheter. It afterwards appeared that it would be necessary to be provided with forceps, longer, more solid, and with larger bowls, than those generally used; for these appeared to me to be unadapted for the grasping and retaining so large a stone.

Being thus prepared, and every thing disposed for the operation, the patient was placed on a horizontal table, and in the usual situation for the operation of lithotomy. The sound, passed into the bladder, confided to an assistant, and maintained in a perpendicular direction, presented its groove under the median line. I raised up the scrotum with the left hand, the cubital margin of which was turned upwards, whilst the thumb and index-finger served to render tense the integuments adjacent to the anus. I preferred to the common bistoury a lithotome analogous to that of Cheselden, mounted on a long handle; and I effected, in the raphe, an incision which commenced at the distance of half-an-inch in front of the anus, and which was prolonged from without inwardly as far as within the intestine. The parietes of this part were sustained, and the posterior surface protected against the point of the instrument, by a wooden gorgeret passed into its cavity, on which the section of the parts was completed. This first step of the operation being terminated, the index-finger of the left hand, passed into the wound, encountered, behind the prostate, the groove of the catheter, and served as a guide to the lithotome, with which an incision as extensive as possible was made, but which was, however, judged to be small in extent. The opening thus made permitted the finger to pass into the bladder, and to come in direct contact with the calculus, which was then raised up, and the canulated sound passed under it. The catheter, being then useless, was withdrawn, and the groove of the sound permitted me to give to the internal incision the extent which was required by the volume of the calculus. I at first attempted to extract this with the common forceps, guided by the sound, but they would not grasp it conveniently: the stone escaped from the instrument, and remained engaged in the wound of the bladder. It was necessary to have recourse to the forceps above designated, by which more considerable and better directed efforts were employed, which were soon followed by the extraction of the stone. Injections of warm water were made into the bladder. But a few drops of blood were lost in the operation. A piece of lint placed between the lips of the external wound prevented its too-prompt union; some compresses, sustained by a T bandage, were then applied, and the patient was carried to his bed.

The stone weighed nine ounces and a half; its figure was a regular oval, the greater diameter of which was three inches and two lines, and the smaller two inches and a half. Some fibres of the transverse muscle of the perineum, the sphincter of the anus throughout its whole extent, the anterior paries of the rectum, and the corresponding part of the bladder, were the parts divided by the instrument. The prostate remained untouched at the front of the wound; the vesiculæ seminales were on its sides: no blood-vessels, no nerves of importance, were injured.

No serious symptoms were manifested after the operation. Some febrile commotion which happened on the second day, and which continued for some time, was so slight that it caused no anxiety. The wound of the sphincter was maintained dilated, in order that the feces might freely pass externally, and not enter the cavity of the bladder. The urine for from fifteen to eighteen days passed wholly by the anus, with the exception of a few drops, which from time to time escaped by the urethra. The prolongation of this accident caused me some anxiety, and induced me to explore the state of the parts. I found that the margins of the wound were hard, tumefied, and in a state of chronic inflammation, which was readily explicable, from the long irritation they had suffered. I first thought of passing a gum-elastic catheter into the urethra; but it appeared afterwards that it would be better to wait until the parts had regained their natural state. After waiting some time longer, without seeing any sensible progress in the cure, I made a new examination of the parts about the wound. I passed a finger into the bladder, and found its internal surface lined, in the parts around the wound, by a great number of adherent and encysted particles of gravel. A little friction of the part detached a portion of false membrane, about an inch in length, in which the gravel was fixed so as not to be separated from it. The same means, repeated several times in the following days, enabled me to extract the remainder of this singular production. The catheter was then introduced: the urine at first passed almost entirely by its canal; but the mucous matter with which the urine was charged soon obstructed it, and it was necessary to have recourse to frequent injections of warm water to maintain it free. Diluent drinks were prescribed; the urine became more limpid; the patient recovered his appetite and strength; the cure made a rapid progress, and seemed to be near its completion.

It should be observed, that no fecal matter passed into the bladder when that matter was solid; but, when the contrary took place, it passed in some degree by the catheter. This accident hardly merits being noticed, because no injury resulted from it, and the urine, when it passed by the wound, carried back into the rectum any solid portion that might have entered the bladder.

The patient could at length get up, and take long walks; and the linen covering the wound was hardly moistened by the urine. Satisfied with his state, and relieved from his pains, he requested permission to return to his family, and set out on the fiftieth day after the operation. The parts were then in the following state. There was no longer any induration or tumefaction of the edges of the wound: this had become diminished to less than half its original size, and its consolidation had been effected from the bottom towards the surface. A month afterwards the patient wrote to me, to say that he had quite recovered his health; that, a few days after he had arrived home, he had ceased to use the catheter; and that his urine was voluntarily expelled, without any portion of it escaping by the anus.

